# Cocoa pod husk, a new source of hydrolase enzymes for preparation of cross-linked enzyme aggregate

**DOI:** 10.1186/s40064-015-1621-3

**Published:** 2016-01-20

**Authors:** Faridah Yusof, Soofia Khanahmadi, Azura Amid, Safa Senan Mahmod

**Affiliations:** Department of Biotechnology Engineering, Faculty of Engineering, International Islamic University Malaysia, P.O. Box 10, 50728 Kuala Lumpur, Malaysia

**Keywords:** Cocoa pod husk, Hydrolase enzymes, CLEA-lipase, Face centered central composite design, Response surface methodology

## Abstract

Cocoa pod husk (CPH) is a by-product of cocoa production obtained after removing the beans from the fruit. The analysis of CPH has shown that it contains high amounts of protein. This study is aimed to utilize this protein source in hydrolase enzyme production. In this study, seven hydrolase enzymes (amylase, fructosyltransferase, mannanase, glucosidase, glucanase, lipase and protease) were screened from CPH for the first time for feasible industrial production. Among these hydrolases, lipase was chosen for the next steps of experiments as it has a lot of applications in different industries. The extraction of high active lipase from CPH has been done under optimum conditions. The condition that was optimum for the three major factors was achieved using Face centered central composite design (FCCCD) with response surface methodology (RSM) to obtain the highest enzyme activity of crude lipase from CPH. The optimum condition of extraction is used for preparation of cross-linked enzyme aggregate (CLEA). For the production of immobilized biocatalyst, the technique of CLEA is considered as an effective technique for its industrially attractive advantages. Referring to the results of OFAT, CLEA-lipase was prepared in the best condition at the presence of 30 mM ammonium sulphate, 70 mM glutaraldehyde with 0.23 mM Bovine serum albumin as an additive. Immobilization effectively improved the stability of lipase against various organic solvents.

## Background

Cocoa pod husk (CPH) is a waste by-product of the cocoa industry, obtained after the removal of the cocoa beans from the fruit. Approximately 52–76 % of the weight of the cocoa fruit is pod husk (Donkoh et al. 1991). For each ton of dry beans produced, ten tons of cocoa pod husks are generated, which presents a serious challenge for waste management (Figuiera et al. 1993). It may be a significant source of disease inocula, such as black pod rot (Figuiera et al. 1993; Barazarte et al. 2008). In 90s, some researchers chemical analysis of CPH showed high percentage of crude protein, between 70 and 90 g/kg (Donkoh et al. 1991; Vriesmann et al. 2012). Since then it is recognised that, extraction of the protein from this CPH has immense economic advantages for the cocoa producer countries and it can reduce some of the environmental problems as well.

Hydrolase is a group of enzymes that can be extracted from the CPH protein. Hydrolases are the most frequently used enzymes in organic chemistry due to several reasons, one being that they are easy to use because they do not need cofactors. Among the hydrolase enzymes, lipases are the most widely used enzyme in the pharmaceutical and drug industry (Chen and Tsai 2000; Gotor-Fernández et al. 2006; Xin et al. 2001), production of biodiesel (Abdelmoez et al. 2013; Raita et al. 2010; Stergiou et al. 2013), laundry detergent (Emtenani et al. 2013; Hasan et al. 2013; Rathi et al. 2001; Sarkar et al. 2012) and food modifications (Aravindan et al. 2007; Undurraga et al. 2001). Lipases are the most relevant enzyme in organic chemistry because they can combine a wide range of substrates with a high regio- and/or enantio specificity and/or selectivity. Lipases are complex enzymes which has a close and open conformation.

The extraction of lipase and lipase activity are influenced by many factors, such as concentration of buffer, pH and the ratio of source. When many factors and interactions affect desired response, response surface methodology (RSM) is an effective tool for optimizing the process. As the needed information about the shape of the response surface is applied, RSM is an effective statistical method that uses a minimum of resources and quantitative data from an appropriate experimental design to determine and simultaneously solve a multivariate equation (Quanhong and Caili 2005). By using the design of experiment (DOE) method, the effect of main factors and their possible interactions can be determined accurately. However, the biological origin, where enzymes are submitted to a strict regulation, makes enzymes quite unstable (Garcia-Galan et al. 2011). A short catalytic lifespan hampers their usefulness and increases the cost of the enzyme-based applications (Stepankova et al. 2013). In general, enzymes are soluble and they can be inhibited by substrate, product and other components (Garcia-Galan et al. 2011). Moreover, the stability of enzymes is insufficient under the processing condition for their use in the industry. Therefore, the immobilization of enzyme is the simplest solution to solve the free enzyme problems. The performance, stability, activity, and selectivity of the enzyme can be improved with immobilization tools. Immobilization improves the control of reaction and avoid product contamination by enzyme (Hartmeier 1985). Immobilization can improve the structural rigidity of the protein and stabilization of the multimeric enzyme, which prevents disassociated-related inactivation and make the enzyme less sensitive to the environment (Guzik et al. 2014; Hernandez and Fernandez-Lafuente 2011; Hwang and Gu 2013). Moreover, this powerful tool enhances the reusability of enzyme for an extended period of time. Enzymes can be immobilized via three major methods: (1) encapsulation or entrapment, (2) binding to support or (3) cross-linking (carrier free).

The preparation of cross-linked enzyme aggregates (CLEAs) is a relatively recent enzyme immobilization technique developed in the group of Prof Roger Sheldon (Sheldon 2011). This method has attracted researches in recent years, because of its many advantages and simplicity. In the CLEA method, enzyme is precipitated to obtain aggregates. In the next step, the aggregate is cross linked with different reagents and it is not a very complex process. The best known reagent for preparation of CLEA is glutaraldehyde (Sheldon 2011). The CLEA technology combines both purification and immobilization into a one-step process and it does not need highly pure enzymes (Hwang and Gu 2013). However, very high levels of enzyme purification should not be expected (Garcia-Galan et al. 2011).

During the immobilization via CLEA, the internal structure of the enzyme can be infiltrated by glutaraldehyde. The aldehyde group reacts with the amino groups of the protein, and the reaction with other groups (thiols, phenols, and imidazoles) may eventually occur (Habeeb and Hiramoto 1968; Migneault et al. 2004; Wine et al. 2007). A chemical aggregation of the enzyme may be produced with the addition of glutaraldehyde to a protein solution which causes a reaction among the protein molecules and can directly yield a “solid biocatalyst” (Barbosa et al. 2014; Caballero Valdés et al. 2011).

Sometimes the amine content of enzyme may be too low to yield effective cross-linking. To overcome this issue, the aggregation can be prepared using certain additives such as Bovine Serum Albumin (BSA) which has a large number of amine groups (Dong et al. 2010). The addition of BSA facilitates the CLEA formation by enhancing the storage, activity and stability of CLEA. The separation of solid biocatalysts, by filtration or centrifugation, is easy and inexpensive (Talekar et al. 2012). In contrast, CLEA is not mechanically resistant and may be considered too soft for some industrial applications. Moreover, the viscosity of some of the precipitants makes the recovery of CLEA difficult and complex on a large scale (Garcia-Galan et al. 2011).

In this work, seven of the hydrolase enzymes were screened from the CPH. Three significant factors (buffer concentration, buffer pH, and ratio of CPH) were considered for the optimization in RSM. Optimum lipase activity is used for the preparation of CLEA-lipase. The individual effects of three selected parameters (concentration of precipitation, cross-linker and additive) were investigated. The structure of prepared CLEA is shown with field emission scanning electron microscopy (FE-SEM).

## Methods

### Materials

Fresh cocoa fruits were collected from the Malaysian Cocoa Board, Jengka, Pahang. *p*-*nitrophenyl palmitate* (*pNPP)* and other chemicals were obtained from Sigma–Aldrich. The Sartorius VivaSpec Spectrophotometer (New York, USA) was used for optical absorbance measurements and Eppendorf centrifuge 5804 (Hamburg, Germany) was used for centrifugation. All experiments were done in triplicates.

### Screening and extraction of hydrolase from cocoa pod husk

Freshly collected chopped CPHs were blended in 100 ml of chilled sodium phosphate buffer for about 25 s. The extract was filtered by gravity through layers of nylon stockings. The filtered extract was centrifuged at 10,000 rpm for 45 min in a cooling centrifuge at 4 °C. The supernatant of this extraction was then stored at −20 °C to be used for further processes.

### Enzymatic assays

#### Lipase activity assay

28 mg of Triton 100-X was dissolved in 100 ml (v/v) plus 1.7 ml of 1 % sodium dodecyl sulphate, while continuously stirring to obtain a *p*-*nitrophenylpalmitate* stock solution. For a reaction to take place, 1 ml of *p*-*nitrophenylpalmitate* stock solution was incubated with 1 ml of 0.1 M Tris–Hcl at pH 8.2 and 0.5 ml purified sample in a water bath for 30 min at 37 °C and 1 ml of 1 M NaOH was added finally to stop the reaction. The measurement of the incubation product, *p*-*nitrophenol*, was taken by the absorbance reading at 410 nm. The absolute amount of *p*-*nitrophenol* produced was calculated from the calibration graph constructed with a known amount of *p*-*nitrophenol*. One unit of enzyme activity is defined as the amount of enzyme required to release 1.0 μmol of *p*-*nitrophenol* per minute, under assay conditions.

#### Protease activity assay

This procedure, described by Sigma-Aldrich, uses casein as a substrate to measure the activity of protease. The casein solution was prepared by dissolving 1 g of casein in 100 ml Tris–HCL buffer at pH 8. The enzyme solution (1 ml) was added to the casein and incubated for 20 min at 35 °C. The reaction is terminated by adding 4 ml of 10 % trichloroacetic acid (TCA). The absorbance was measured at 280 nm and a tyrosine standard curve was prepared. One unit of enzyme activity is defined as the amount of enzyme required to hydrolyse casein to produce colour equivalent to 1.0 μmol of tyrosine per minute, under assay conditions.

#### Amylase activity assay

Amylase activity was assayed with 1 % (w/v) starch in 50 mM sodium phosphate buffer as the substrate, at pH 7.0. The reaction mixture contained 2.5 ml sodium phosphate buffer (50 mM, pH7.0), 2.5 ml starch solution and 1 ml of sodium chloride. These mixtures were incubated at 37 °C for 10 min for pre-treatment preparation. After that, 1 ml distilled water and 0.5 ml crude enzyme were added and the reaction mixtures were then incubated at 37 °C for 15 min followed by the addition of 0.5 ml sodium hydroxide (1 M) and 0.5 ml of dinitrosalicylic acid (DNS) reagent to stop the reaction. The mixture was boiled for 10 min and then cooled immediately. The optical densities of the mixture were estimated at 540 nm in UV spectrophotometer. The definition for a unit of enzyme activity is given as the quantity of enzyme that is needed for the release of 1 µmol of reducing sugar that equals to glucose per minute under standard assay conditions.

Table 1 summarizes the preparation of several other enzymatic assays. For these cases, the quantity of reducing sugars released was measured using the DNS method. The definition for a unit of enzyme activity is given as the quantity of enzyme that is needed for the release of 1 µmol of reducing sugar that equals to glucose per minute under standard assay conditions.

**Table 1:** Preparation of several enzymatic assays

Activity assay	Assayed with	Reaction mixtures	Incubation
Mannanase	0.5 % (w/v) locust bean gum (LBG) in 50 mM sodium citrate buffer at pH 4.8	The enzyme preparation (0.2 ml) was added into 1.8 ml of substrate.	At 50 °C in the water bath for 5 min
Glucanase	1 % (w/v) carboxymethylcellulose (CMC) in 50 mM sodium citrate buffer at pH 4.8	The enzyme preparation (1 ml) was added into 1 ml of substrate and 1.0 ml of sodium citrate buffer	At 50 °C in the water bath for 30 min
Glucosidase	15 mM cellobiose in 50 mM sodium citrate buffer at pH 4.8	The enzyme preparation (0.2 ml) was added to 0.2 ml of substrate and 0.2 ml of sodium citrate buffer	At 50 °C in the water bath for 15 min
Fructosyltransferase	40 % (w/v) sucrose in 50 mM sodium acetate buffer at pH 5.4	The enzyme preparation (1.0 ml) was added into 49 ml of substrate	At 50 °C in the water bath for 5 min

### Protein concentration

Protein content of the extraction was determined by the Bradford method (BRADFORD, 1976) with a standard of BSA and scanning by Magellan Data Analysis Software at the absorbance wavelength of 595 nm.

### Experimental design for the optimum extraction of CPH

The experiment was designed to optimize the condition parameters for the extraction of CPH that yield the highest enzyme activity. Three significant parameters were tested in this design. First, varying the buffer concentration (50, 100, 150 mM); second, the buffer pH (6, 7, 8) and third, varying the ratio of CPH (5, 6, 7 w/v). The RSM involving three levels of FCCCD was used to optimize these components for enhanced enzyme activity. A total of 20 runs were employed with six replicates at the centre point. Table 2 shows the coded and actual values of the variables at different levels. The statistical software package Design Expert (version 6.0.8) was used to analyze the result.

**Table 2:** Experimental design using FCCCD of three independent variables with their actual and coded values and six centre points showing the experimental and predicted response

	pH	Conc. (Mm)	Ratio (%w/v)	Lipase activity (U/ml)	
				Experimental	Predicted
1	7.00 (0)	100.00 (0)	6.00 (0)	6.82	6.17
2	8.00 (+1)	150.00 (+1)	5.00 (−1)	7.84	7.61
3	6.00 (−1)	50.00 (−1)	7.00 (+1)	8.62	9.18
4	8.00 (+1)	150.00 (+1)	7.00 (+1)	8.03	8.61
5	7.00 (0)	100.00 (0)	6.00 (0)	6.79	6.17
6	8.00 (+1)	100.00 (0)	6.00 (0)	9.23	8.98
7	8.00 (+1)	50.00 (−1)	5.00 (−1)	10.02	10.59
8	6.00 (−1)	150.00 (+1)	7.00 (+1)	9.01	8.91
9	8.00 (+1)	50.00 (−1)	7.00 (+1)	11.43	11.26
10	7.00 (0)	100.00 (0)	6.00 (0)	5.97	6.17
11	7.00 (0)	100.00 (0)	6.00 (0)	6.82	6.17
12	6.00 (−1)	100.00 (0)	6.00 (0)	7.48	7.58
13	6.00 (−1)	50.00 (−1)	5.00 (−1)	7.93	7.49
14	7.00 (0)	100.00 (0)	5.00 (−1)	4.93	4.89
15	7.00 (0)	100.00 (0)	7.00 (+1)	6.70	6.24
16	6.00 (−1)	150.00 (+1)	5.00 (−1)	6.58	6.89
17	7.00 (0)	150.00 (+1)	6.00 (0)	6.84	6.50
18	7.00 (0)	100.00 (0)	6.00 (0)	6.47	6.17
19	7.00 (0)	50.00 (−1)	6.00 (0)	8.28	8.13
20	7.00 (0)	100.00 (0)	6.00 (0)	5.84	6.17

### Preparation of CLEA

CLEA lipase was prepared using a modified procedure by (Lopez-Serrano et al. 2002). The enzyme solution from CPH (0.5 ml) was poured into a 15 ml Falcon tube. The final volume of 4 ml solution was prepared by simultaneously adding varying amounts of 50 % saturation of ammonium sulphate, varying amounts of glutaraldehyde solution, and also varying amounts of bovine serum albumin (BSA) as an additive to the enzyme solution. The solution was agitated at 200 rpm for 17 h at room temperature. Then 3 ml of water was added and the mixture was centrifuged at 4000 rpm at 4 °C for 30 min. The supernatant was poured out and the residue was washed with water 3 times, then it was centrifuged and decanted. The final CLEA preparation was kept in 5 ml of water to measure the activity assay of CLEA lipase.

### SDS-PAGE analysis

The lipase preparation was analysed by sodium dodecyl sulphate (SDS)-PAGE. SDS-PAGE was performed according to Laemmli’s method (Laemmli 1970) using gels of 12 % polyacrylamide and a concentration zone of 4 % polyacrylamide. The gel was stained following the silver staining method. A low molecular weight marker was used (16–114 kDa).

### Stability test

The stabilities of the free lipase and its CLEA in the presence of different hydrophilic organic solvents were compared. Residual activity for free and immobilized lipase was obtained by incubating enzyme in substrate-free buffer at various hydrophilic organic solvents (methanol, dioxane, acetone) with different concentrations. After 30 min the substrate was added and the mixture incubated at optimum temperature for more 30 min.

### Structural characterization by field emission scanning electron microscopy (FE-SEM)

Morphology of the prepared CLEA was examined using FE-SEM. The field emission scanning electron micrographs (FE-SEMs) of CLEA were obtained on JSM-6700F (Germany) FE-SEM operated at 5 kV. Sample was freeze dried by rotter freeze dryer, placed on a sample holder, coated with gold before being scanned under vacuum.

## Results and discussion

### Extraction of hydrolase from CPH

Optimum pH and buffer concentration are important factors for the extraction of protein. The optimum pH for the highest protein activity is different in various samples. The highest lipase activity from olive fruit has been extracted at pH 5 (Panzanaro et al. 2010). In prior investigations, the optimal pH values for protein extraction from Mung beans and Red beans were 9 (Wang et al. 2011) and 8.8 (Liu et al. 2011), respectively. The optimal conditions for protein extraction from red pepper seeds and the highest enzyme activity were achieved at pH 8.8 (Firatligil-Durmus and Evranuz 2010). These variations indicate that the activity is more sensitive to pH range. In general, the enzyme activity increases when the pH of the medium is higher than 7.0 (Lv et al. 2008). Temperature is another crucial factor during the extraction in order to maintain proteins’ shape, activity, and stability. To minimize protein denaturation as well as the autolysis of enzymes, the sample must be kept at (0–4 °C) during the process.

Following the Bradford assay, the protein concentration of the crude enzyme mixture is 358 µg/ml. It was observed that the colour of the assay was stable blue and in gradient based on the concentration of BSA in the preparation of the standard curve. The dark blue sample indicated the presence of active proteins and that the reaction has occurred between the protein and dye.

**Fig. 1:**
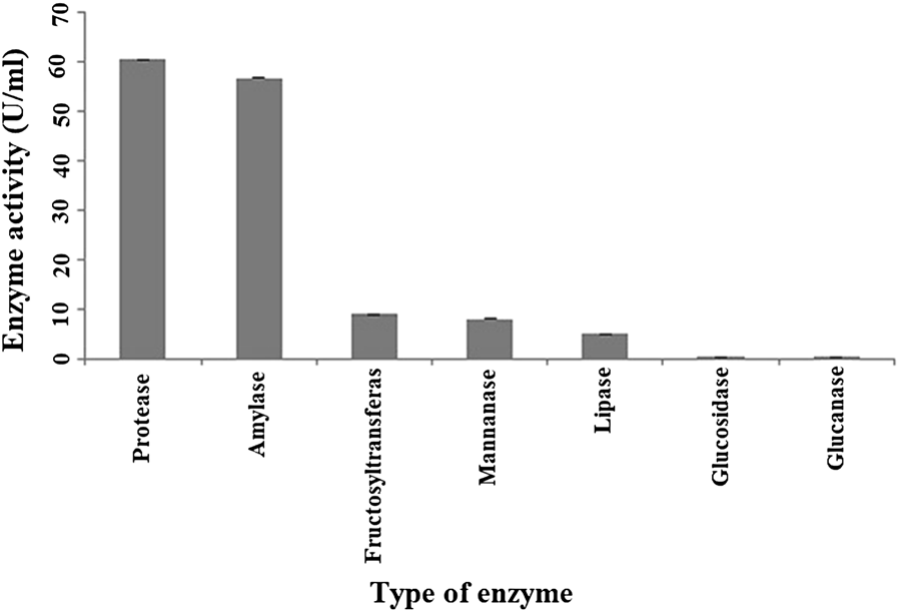
Measuring the activity of various hydrolases from CPH

Figure 1 shows the screening of hydrolase from CPH and each the enzymatic assay performed. The highest enzyme activity was obtained from Protease (60.5 U/ml). The enzyme activity for amylase, fructosyltransferase, mannanase, lipase, glucosidase, and glucanase were conducted at (56.7, 9.04, 8.1, 5, 0.4, 0.3 U/ml), respectively. Among these hydrolases, lipase was chosen for the next steps of the experiments due to its high efficiency in various industrial applications.

### Optimization of CPH extraction

Lipase production, increased by several folds, show that experimental designs based on statistics is important in optimizing the medium. According to (Wu et al. 2007), this method has several advantages such as establishing the effects of the factors as well as building a system model that has less experimental requirements. Following the OFAT experiments, optimal conditions for the three important factors (concentration, pH, and ratio) were obtained using Face Centered Central Composite Design (FCCCD) under RSM. Table 2 shows the experimental and predicted values of lipase activity acquired from the regression equation of 20 combinations in each run. From the results, the highest amount of lipase produced from CPH (11.43 U/ml) was observed in run 9 under 7 % ratio of CPH and 50 mM sodium phosphate buffer at pH 8. The lowest was observed in run 14 (4.931 U/ml), where 5 % CPH is used with 100 mM buffer at pH 7. This proves that the lipase production is better with the design matrix of FCCCD compared to OFAT and screening. The results reported a 2.5-fold increase in the lipase production by using RSM.

A second order regression equation revealed the dependence of the lipase activity from CPH on the medium constituents. The parameters of the equation have been obtained by multiple regression and the variables that were screened were expressed in terms of second-order polynomial equation as follows:

$$ \begin{aligned} {\text{Y}}\left( {{\text{lipase activity U}}/{\text{ml}}} \right) & = \left( { + 6. 4 7+ 0. 6 9 {\text{A}} - 0. 80{\text{B}}} \right) \, + \, \left( {0. 6 5 {\text{C}} + 1. 8 5 {\text{A}}^{ 2} + 1.0 6 {\text{B}}^{ 2} } \right) \, \\ & \quad - \, \left( {0. 6 9 {\text{C}}^{ 2} - 0. 5 8 {\text{AB}} - 0. 1 9 {\text{AC}} + 0.0 6 5 {\text{BC}}} \right), \\ \end{aligned} $$

where response (Y) is the activity of lipase. A, B, and C represent the buffer pH, buffer concentration, and ratio of CPH, respectively.

The analysis of variance (ANOVA) was conducted to verify the adequacy of the model and Fisher’s statistical analysis was used to test it. The results are recorded in Table 2. The F value of 20.55 and *p* value of <0.0001 from this model provides a significant inference. A p value less than 0.05 is considered significant. The efficiency of the model is shown with a higher value of R^2^ (0.9487) and adjusted R^2^ (0.9025). The R^2^ value should be maintained within the range of 0–1.0. As the value gets closer to 1.0, the model is considered to be a better fit (Reddy et al. 2008). The signal to noise ratio is measured by adequate precision, where a ratio greater than 4 is considered a requirement for desirable models. The ability of the model to navigate the design space is indicated by the adequate precision value of 17.618 for lipase activity.

Table 3 lists coefficient values of the regression equation. The significance of each coefficient are determined using the *p* values, which is also an indication of the strength of interaction between each variable that is independent; as the *p* values get smaller, the significance of the corresponding coefficients get bigger. All the three linear coefficients (A, B, and C), interaction term AB (pH and concentration), and all the three quadratic coefficients (A^2^, B^2^ and C^2^), were found to be significant with (p < 0.05) and affects the overall production remarkably.

**Table 3:** Analysis of variance of quadratic model for lipase production

Source	Sum of squares	F value	*p* value
Model	43.16	20.55	<0.0001
pH, A	4.80	20.58	0.0011
Concentration, B	6.37	27.29	0.0004
Ratio, C	4.21	18.05	0.0017
A^2^	9.42	40.37	<0.0001
B^2^	3.07	13.14	0.0047
C^2^	1.31	5.60	0.0396
AB	2.67	11.43	0.0070
AC	0.29	1.24	0.2920

Literature has documented several fold increases in the production of lipase using RSM (Aybastıer and Demir 2010; Dwevedi and Kayastha 2009; Khoramnia et al. 2010; Muralidhar et al. 2001; Ruchi et al. 2008). For example, a 20 % increase in the production of lipase was achieved under optimal conditions following the use of RSM (He and Tan 2006).

**Fig. 2:**
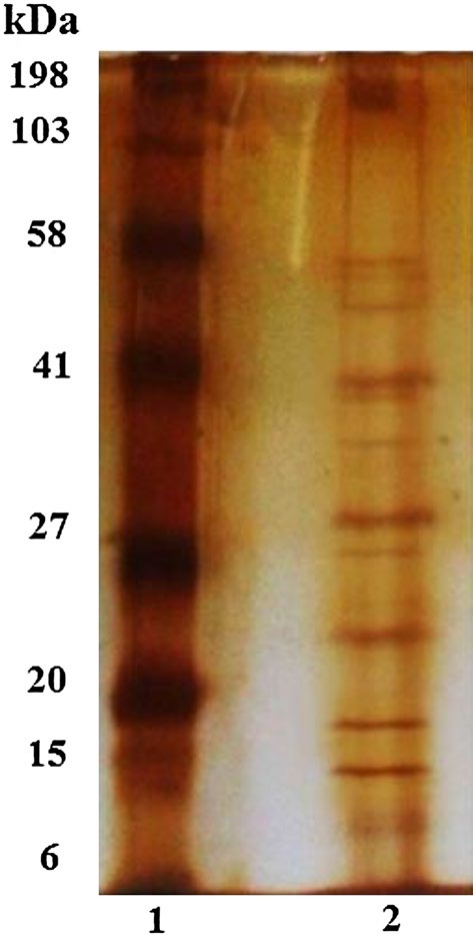
SDS-PAGE of lipase extracted from CPH. *Lane 1* molecular weight marker, *Lane 2* molecular weight of lipase

Sodium dodecyl sulphate SDS-PAGE was used to obtain the protein profile and to estimate the molecular weight of the lipase extracted from the CPH. The molecular weight of the lipase has been estimated 40 kDa in rice bran (Bhardwaj et al. 2001), 24 kDa in buckwheat seed (Suzuki et al. 2004), and 55 kDa in rapeseed (Sammour 2005). These studies reveal that enzymes have different molecular weights depending on their sources. In silver staining, more than one band is resulted from the SDS-PAGE analysis, which indicates that the extract of the CPH contains many proteins of different sizes. Since the sample was only precipitated, all the proteins’ bands were clearly shown and possibly one of them belongs to lipase (Fig. 2). Additionally, the thickness of the band indicates the abundance or the intensity level of the protein.

### Study of operating conditions of extraction of CLEA-lipase from CPH (OFAT analysis)

In this study, the individual effect of three selected parameters (concentration of ammonium sulphate, glutaraldehyde, and BSA) were investigated to determine the highest enzyme activity. Production of CLEA-lipase from CPH was carried out in 15 ml falcon tubes. The content of the tube was made up of 0.5 ml crude lipase from CPH, (10–50 % w/v) saturated ammonium sulphate, (40–80 mM) glutaraldehyde and (0–0.37 mM) BSA and all reactions were allowed to proceed for 17 h.

**Fig. 3:**
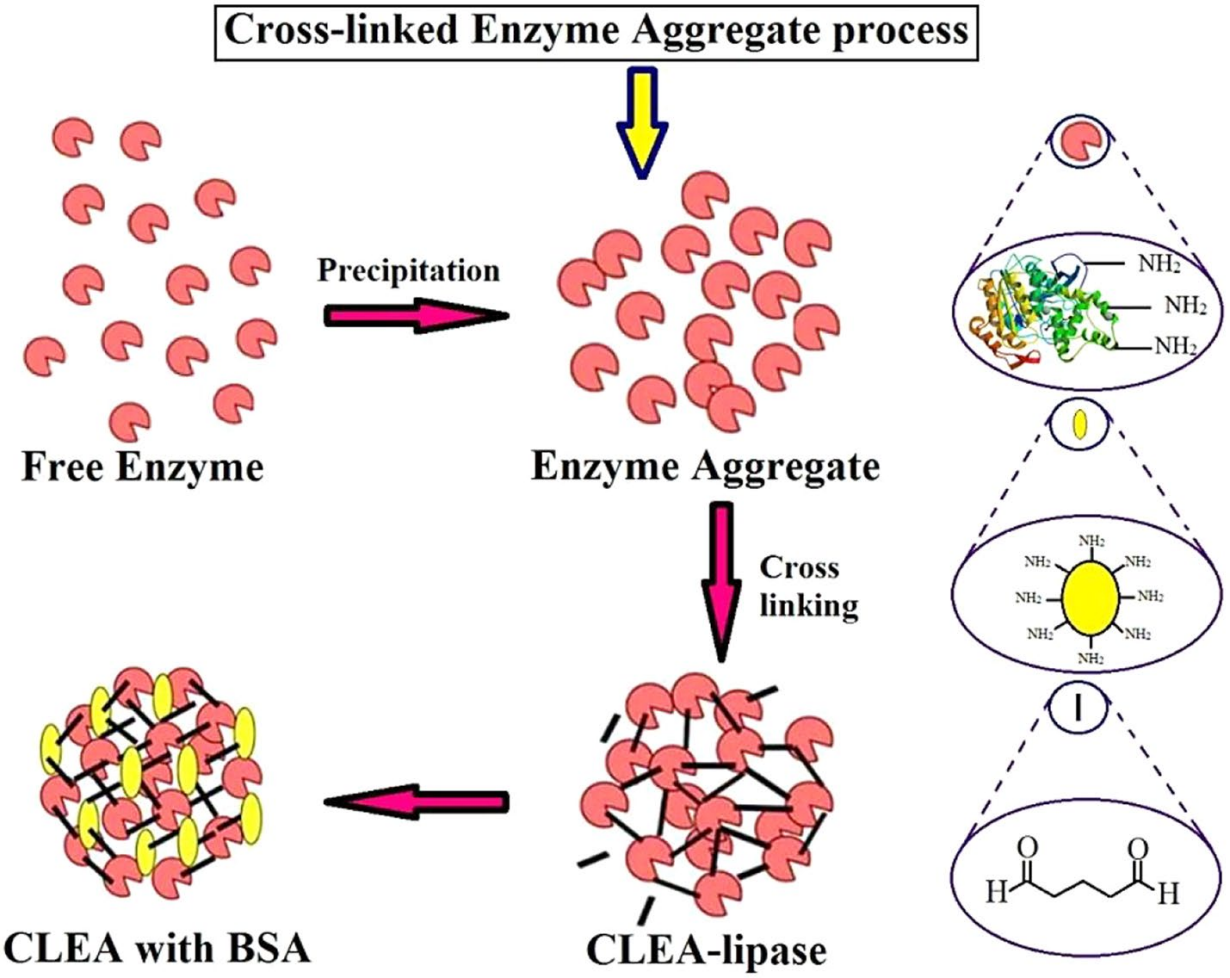
Schematic picture of CLEA preparation with addition of BSA as additive

Among the tested values, 30 % (w/v) ammonium sulphate, 70 mM glutaraldehyde, and 0.23 mM BSA were found to be optimum values for the production of CLEA-lipase. Figure 3 shows the formation of CLEA with the addition of BSA as an additive.

The final particle size of CLEA depends on the type and amount of the precipitant, stirring rate, concentration of glutaraldehyde, and protein concentration (Garcia-Galan et al. 2011; Yu et al. 2006). In order to capture the enzyme activity in the final product of CLEA, the entire free enzyme should be precipitated to a significant level. Addition of organic solvents, salts, non-ionic polymers or acids can aggregate and precipitate the enzyme. Among different precipitants, ammonium sulphate has worked the best for precipitating all the free enzyme activity (Talekar et al. 2012; Yu et al. 2013). The amount of ammonium sulphate plays a significant role in controlling the enzyme activity and particle size formation. It was noted that the maximum activity of the insoluble cross-linked enzyme was detected when most of the protein has been precipitated out of the solution and vice versa (Yu et al. 2006).

**Fig. 4:**
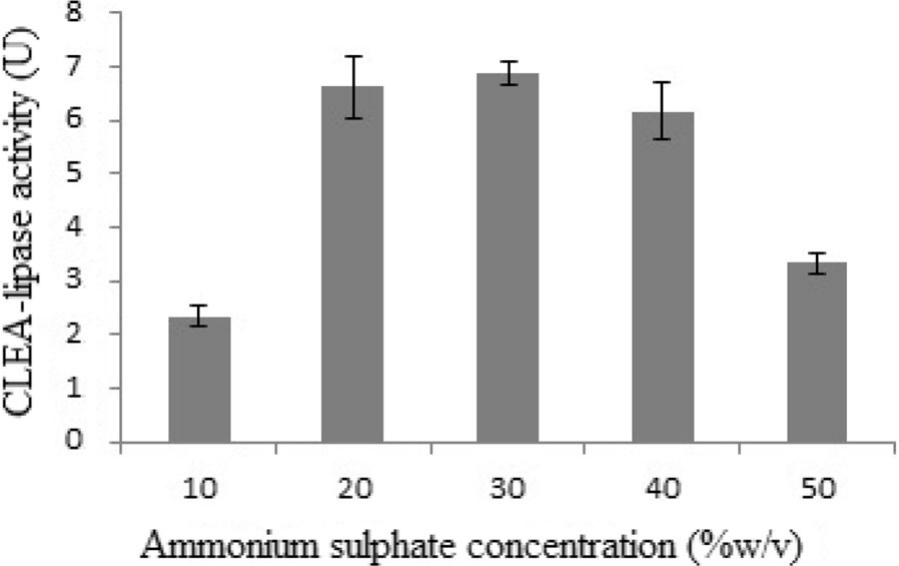
Effect of different concentration of ammonium sulphate (10–50 %w/v) on CLEA-lipase activity from CPH

The production of CLEA-lipase from CPH was amplified with the increase of ammonium sulphate concentration. The observed increase in the production of CLEA-lipase was between 10 and 30 % (w/v) ammonium sulphate. However, further increase in the concentration rather decreased the production (Fig. 4). High concentrations of ammonium sulphate can decrease the enzyme activity and reduce the protein residues in the supernatant. This may result in too little precipitant to form the aggregates and indicates that additional free lipase was forming insoluble enzyme aggregates. High ammonium sulphate concentration may lead to protein denaturation that is responsible for the loss of activity (Wang et al. 2011).

The substrate concentration, below the requirement for the enzyme saturation, allows the enzyme environment to partition the substrate toward the enzyme. This results an increase in the enzyme activity.

**Fig. 5:**
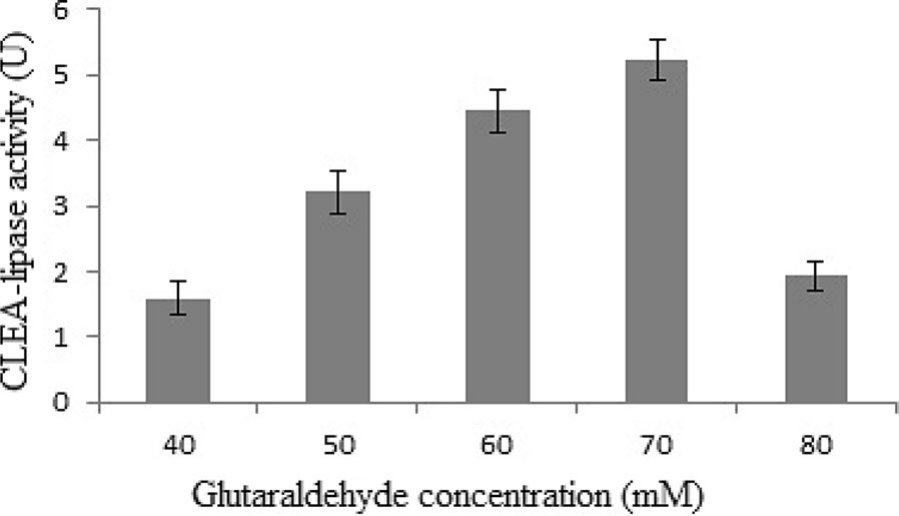
Effect of different concentration of glutaraldehyde (40–80 mM) on CLEA- lipase activity from CPH

The level of glutaraldehyde concentration for maximal lipase activity was determined by testing different levels of glutaraldehyde with the concentration of 40–80 mM. The highest lipase activity of 5.23 U (Fig. 5) was observed at approximately 70 mM concentration of glutaraldehyde. In general, the reaction of glutaraldehyde molecule with amine residues of the lipase surface can impact (positively/negatively) the lipase activity (Guauque et al. 2014).

The concentration of glutaraldehyde has significant effects on the enzyme activity. In the presence of low amounts of cross-linkers, the enzyme molecule will leach into water, because the immobilized enzyme is still flexible and unstable. An excessive amount of GA can decrease the enzyme activity due to loss of the minimum flexibility needed for the activity (Sheldon 2011). Zhu and Sun (2012) reported that this reaction could be due to more multi-point chemical bonds between enzyme molecules and membrane surface at a high GA concentration.

The internal aldehyde group of glutaraldehyde attached with primary Ɛ-amino group from lysine and the result of this reaction is the Schiff bases (Barbosa et al. 2014).

Sometimes CLEA may not be effective due to low enzyme Lys residue, since the cross linking involves reaction of amino groups of Lys residues at the external surface of the enzyme (Talekar et al. 2012). In order to enhance the activity of enzyme, a second protein like BSA with a large amount of Lys can be used for the preparation of CLEA. The addition of BSA can prevent the formation of clusters in the excess concentration of glutaraldehyde, which leads to the limitation of mass transfer. Moreover, the addition of BSA provides lysine residues and makes positive impact by creating bonds with glutaraldehyde to prevent the denaturing of the targeted protein. However, the BSA decreases the volumetric loading of the target enzyme, since the inert protein occupies a portion of the volume (Barbosa et al. 2014; Rodrigues et al. 2014).

**Fig. 6:**
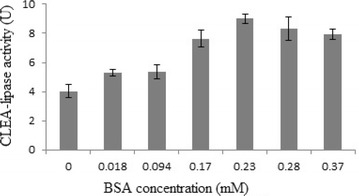
Effect of different concentration of BSA (0–0.37 mM) on CLEA-lipase activity from CPH

Figure 6 shows the effect of different concentrations of BSA as additives (0–0.37 mM) on the CLEA-lipase activity. A rise in the CLEA-lipase production was seen at levels ranging from 0 to 0.23 (mM) BSA, where a decrease in the production was observed with subsequent increase in concentrations. This may be due to leaching of extra protein in the media.

The addition of 10 mg BSA in the formation of CLEA of aminoacylase increased the activity to 82 %, which was only 24 % before the addition (Dong et al. 2010).

The lower activity of CLEA lipase of CPH (9 U) compared to the crude enzyme (11.26 U/ml) may be due to the hydrophobic and rigid structure of the enzyme after cross linking. Therefore, it is hard for the enzyme to accept a large substrate like *pNPP*. The immobilization of the enzyme can block the active site of the enzyme (Iyer and Ananthanarayan 2008; Mateo et al. 2007; Sheldon 2007) and may also increase the diffusion problems (Gottifredi and Gonzo 2005). These issues have led to the reduction of enzyme activity after the immobilization. The enzyme can denature and decrease in activity after solubilising in the immobilization medium.

After the formation of CLEA, the excess glutaraldehyde was removed by repeatedly washing in water to avoid any modification of protein by the free glutaraldehyde. However, the already reacted glutaraldehyde may continue the cross linking process with the protein even after the washing steps.

### Stability of CLEA-lipase against hydrophilic organic solvents

**Fig. 7:**
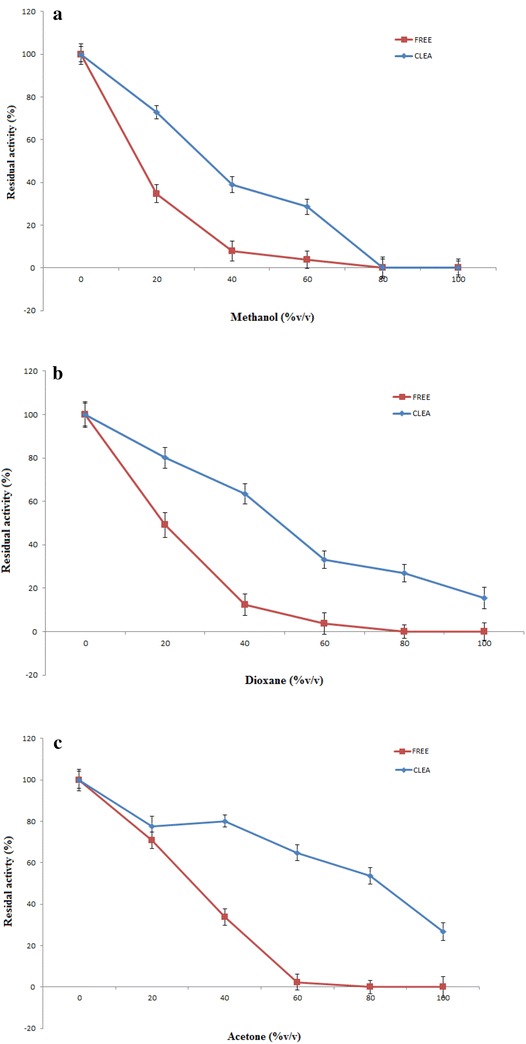
Comparison in the resistance of both the free lipase and its CLEA against hydrophilic solvents. Free lipase and CLEA were incubated at 45 and 60 °C, respectively, for 30 min in various buffer/solvent mixtures: methanol (**a**), dioxane (**b**), and acetone (**c**), and their residual activities were determined. A value of 100 % refers to the activity obtained by the enzyme in the solvent-free buffer solution

In our previous work, the CLEA-lipase from CPH was characterized and the results indicated that the immobilized lipase has superior stability in response to different ranges of temperature (25–60 °C) and pH (5–10) in comparison to free form of the enzyme (Khanahmadi et al. 2015). In this experiment, the stability of CLEA-lipase against hydrophilic organic solvents (methanol, dioxane and acetone) was studied. The optimum temperatures for enzymatic reaction of immobilized and free lipase were found to be 60 and 45 °C, respectively. Organic solvents are known to have detrimental effect on the enzyme action. Based on Fig. 7a, the stability of both forms of lipase were low in the presence of methanol. However, CLEA was relatively more stable against methanol at a higher level. In the presence of 60 % (v/v) methanol, the free lipase retained only 4 % of its initial activity, whereas for CLEA, it was 29 %. When solvent concentrations of both dioxane (Fig. 7b) and acetone (Fig. 7c) were increased up to 100 % (v/v), a gradual reduction in the activity was observed for both enzyme forms comparatively within a range (0–100 %, v/v). CLEA exhibited a higher retention in activity than the free enzyme. A similar trend of stability was too observed in the other study (Xu et al. 2011).

There are several factors responsible for the effects of organic solvents. The organic solvents mostly cause denaturation of proteins mainly due to the disruption of intra non-covalent interactions. In other cases, organic solvents can act either as an inhibitor or a substrate itself rendering competitive inhibition or substrate inhibition. Organic solvents can interrupt intra-molecular hydrogen bonding that stabilizes the tertiary structure of any proteins. Apart from that, the organic solvents change the physical properties such as dielectric constant, polarity, and hydrophobicity. This change affects the solvation of substrate and/or transition states, and hence interferes with their binding to enzyme molecule (Mozhaev et al. 1989).

High stability of immobilized lipase can be attributed to the structure of the immobilized enzyme that becomes rigid because of the cross-linking. The immobilization is a useful approach to protect the enzyme from the detrimental influence of organic solvents. This is achieved by suppressing the tendency of the enzyme towards unfolding and is accompanied by the loss of tertiary structure necessary for the activity.

The absence of water in the incubation solution can play a role, since increased stability of enzymes under low moisture conditions has already been proven. It has been shown that dehydration can exorbitantly retard the thermal inactivation of enzymes, which preserves their conformational rigidity (Volkin et al. 1991). Although rigid structure of CLEA maintains higher retention at higher concentration of dioxane and acetone, the result was contradictory for pure methanol solvent known to cause severe denaturation in various enzymes (Herskovits et al. 1970).

### Structural characterization of CLEA lipase from CPH by Field Emission Scanning Electron Microscopy (FE-SEM)

The shape and size of CLEA particle is still relatively unknown. The final particle size of CLEA depends on the rate of stirring, rate of precipitant addition, concentration of protein, and precipitation time. CLEA has a small pore size that reduces the substrate diffusion rate. Therefore, the proposed methods will allow adequate enzyme volumetric activities even when the substrate is unsuitable for the enzyme. In the case of highly suitable substrate, the enzyme activity will most likely overtake the substrate to drop the substrate concentration inside the particle (and also a pH gradient if H^+^ is consumed or produced by the reaction) (Garcia-Galan et al. 2011). During the immobilization, enzyme solubility decreases in the surrounding medium. While aggregation is slow, the extreme force applied on the structure of the enzyme can cause denaturation. The enzyme structure can be retained if protein molecules are found in time to surround the enzyme. The aggregation speed can be increased to recover a poor enzyme activity. The molecule diameter is set by the surface tension of the aggregate and the hydrophobicity of the surface will control the structures of protein aggregates. As stated by the classical nucleation theory, the nucleus size is controlled by the interaction of the free energy which is either increased by interfacial surface formation or decreased in solid formation. The size of the primary particles in the final aggregate is likely to be small and controlled by the ratio of nucleation and growth.

**Fig. 8:**
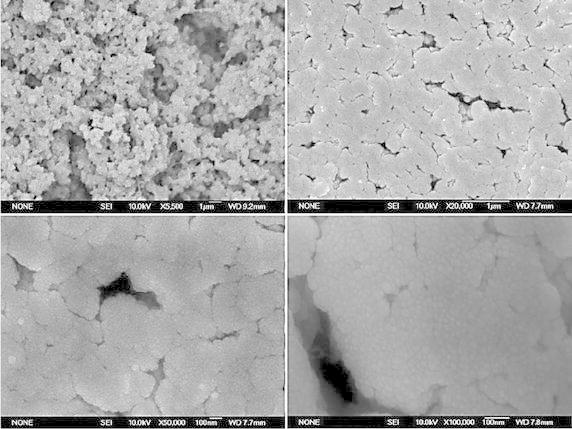
FE-SEM picture of CLEA-lipase from CPH

Field emission scanning electron micrographs revealed that the CLEA from CPH has a spherical appearance. With regard to CLEAs, Schoevaart et al. (2004) reported that CLEAs have either a spherical appearance (Type 1) or a less-structured form (Type 2). Therefore, the CLEA of CPH was the Type 1 aggregates (Fig. 8). The Type 1 aggregates can accommodate a thousand times more enzyme molecules than the Type 2. Unlike the free protein, the enzyme molecules are packed in a small volume, where mass-transport limitations will surely be expected, especially with fast reactions. This effect is found to be small when the CLEA is dispersed in the solution at the end of the cross-linking process.

## Conclusions

In this project, seven of the most important hydrolase enzymes from an agro waste by-product, CPH have been screened for the first time successfully. Lipase is chosen as the enzyme for further experiments because of its wide use in industrial applications. The extraction of Lipase with three significant factors is optimized with FCCCD under RSM. From 20 runs, the highest lipase activity (11.43 U/ml) was achieved at the presence of 50 mM sodium phosphate buffer pH8 with the ratio of 7 % (w/v) of CPH. The results have shown approximately a 2.5 fold increase in the lipase production after optimizing via RSM. The optimum extraction condition was used to prepare the immobilized enzyme through the cross-linked enzyme aggregate. To achieve the immobilization of lipase from CPH, the use of 30 mM ammonium sulphate as precipitate, 70 mM glutaraldehyde as the cross-linker, and 0.23 mM BSA enhanced CLEA properties. CLEA-lipase has shown more stability against hydrophilic organic solvent. CLEA was characterized with FE-SEM to visualize the final structure and determine the shape and type of the CLEA.
